# Examining the Psychological State Analysis Relationship Between Bitcoin Prices and COVID-19

**DOI:** 10.3389/fpsyg.2021.647691

**Published:** 2021-03-22

**Authors:** JianPing Hou, Jingyi Liu, YingJiang Jie

**Affiliations:** School of Economics and Management, Xi’an Technological University, Xi’an, China

**Keywords:** COVID-19, Bitcoin prices, psychological state, financial development, business sustainability

## Abstract

The rapid worldwide spread of COVID-19 forced many countries to enforce complete lockdown and strict quarantine policies. The strict lockdown and quarantine affect the psychological state of people toward cryptocurrency. The current research aims to examine the effect of COVID-19 on Bitcoin prices concerning cumulative deaths and confirmed cases. The research comprises daily data from January 20, 2020, to April 30, 2020, during the initial worldwide breakout of COVID-19. This research employed the augmented Dickey-Fuller test to check the stationarity of data, the co-integration test for the interdependency of variables, and the vector error correction model for identifying the direction and long or short-run relationship between Bitcoin prices and COVID-19. The research results show that Bitcoin prices are negatively significant and related to COVID-19 in the short-run. A unidirectional relationship between Bitcoin prices and cumulative deaths is also observed. Investors and the public’s psychological state were positively significant to Bitcoin prices in the long-term because of cashless transactions, unbanked, and less risky virus traveling. The second reason behind the positive psychological relation is un-centralization and easy-to-make payments by Bitcoin. This study’s finding provides timely evidence to decision-makers on Bitcoin price volatility and its impacts on the public’s psychological states regarding COVID-19.

## Introduction

A new type of financial asset named “cryptocurrency” has been introduced recently. Bitcoin is the dominant element of the cryptocurrency market, a growing and highly volatile market (Bariviera, 2017). The pseudonymous author Satoshi Nakamoto introduced this virtual currency in 2008, and it was traded openly in 2009 (Nakamoto, 2008). Cryptocurrency is a revolutionary addition to electronic trade and exchange. The number of cryptocurrencies is increasing daily, and at present, it is over 5500. Bitcoin is vital in all cryptocurrencies, as it provides a fast, secure, and inexpensive exchange medium to its users.

Bitcoin is destroying the scarcity, which is a prerequisite for ascribing value to any form of money and protects the money from counterfeiting (Buchholz et al., 2012; Böhme et al., 2015). The money’s feature scarcity preserved the legal rules and regulations and ensured the transactions’ accountability records were correct. The central bank controls the money circulation and adjusts the quantity of money in circulation to control its inflation and deflation. Against the backdrop of fiat money, Bitcoin is un-centralized and only tracks the coin holder, which is more complex than the classical bookkeeping system. As peer-to-peer money, Bitcoin issues new currency to the holder of the coin (main private party) as an incentive to maintain the record of transactions, bookkeeping, and verification of the transaction. With all its disadvantages, Bitcoin occupies a huge part of every economy. The price formation of Bitcoin is not up to standard theories of economics like conventional money based on the future cash-flow model, purchasing power parity, and demand-supply featuring (Kristoufek, 2013). The speculative bubble behavior and detachment from macroeconomic determinants are observed in Bitcoin supportive contents. The demand and supply market forces played Bitcoin price dominators (Bouoiyour and Selmi, 2015). As the global financial market got influenced by stock exchange indices, interest rate, exchange rate, inflation, and oil prices, the long-run Bitcoin prices fluctuated due to the volatility of the Dow Jones index and the euro-dollar exchange rate (Van Wijk, 2013; Stavroyiannis, 2018; Su et al., 2018; Li et al., 2019; Sarfraz et al., 2020a).

### Bitcoin Volatility and Psychological Impact of COVID-19

COVID-19 affected all aspects of life, Bitcoin being one of them. The investors’ psychological state has been affected by the pandemic breakout. Bitcoin prices were positively impacted by the coronavirus breakout initially and then increased again. The strict quarantine policies caused an upsurge in online shopping and technology-based life (Azam et al., 2020; Su et al., 2020). Su et al. (2020) examined the Chinese and Italians’ psychological state using the Weibo users sample in Wuhan, China, and the Twitter users in Lombardy, Italy, for two weeks after lockdown. Their psycholinguistic behavior was examined using simplified Chinese and Italian language inquiry, word count, and a Wilcoxon test. The results of Su et al. (2020) revealed that people stayed at home, and due to the shopping applications and other options, they were significantly less stressed. Bitcoin also boosts its exchange rate and effortless shopping during COVID-19 as available online money. Bitcoin positively influenced social encouragement, cybersecurity, and government regulations, and infrastructural quality support on behavioral intentions. This positive relation was observed by utilizing the Bitcoin qualitative trading data of Indonesia with the implication of inner model, outer model, and hypothesis assessment. Moreover, the improvement and enhancement of Bitcoin trade’s quality with developed and more secure transaction process are discussed (Van Wijk, 2013; Vidal-Tomás and Ibañez, 2018; Putra and Darma, 2019; Sarfraz et al., 2020b). Large firms prefer to adopt corporate social responsibility activities’ (Sarfraz et al., 2020c).

Bitcoin is a payment mode with two types of constituencies, i.e., the user of Bitcoin and the quick profit-seekers (Miner) or infrastructure’s maintainer. By employing a simple economic model, the Bitcoin payment system is captured. The limited system output is generated by the congestion queuing game, while the system’s equilibrium is derived by transaction fee and infrastructure level (Huberman et al., 2017). The modern era also demonstrated the rapid increase in Bitcoin usage as the electrical vehicle (EV). The divergent applications are designed during COVID-19 to avoid cash payments. These applications are concentrated on payment security issues as privacy-preserving transactions among suppliers and customers. The Bitcoin payments are considered a more secure payment that eliminated all security issues by the Bitcoin holder’s permission and signature (Erdin et al., 2018).

According to economists, Bitcoin plays a positive role during the pandemic. The pandemic’s nature declared that the virus could be transmitted from person to person. The government enforced cashless money after it was apparent that conventional money was one way the virus was transmitted. The shortage of conventional money, its circulation inefficiencies, and the worsened cashless transactions dilemma attracted cryptocurrency under specific situations. Approximately 1.7 billion non-banking adults faced the fee transfer problem, while more than half of the issues were resolved through a crypto-asset during the pandemic. The sense of safety changes people’s psychological behavior towards Bitcoin. Social media plays a vital role in observing individuals’ reactions, opinions, and mental health who use divergent social media platforms for information (Lima and De Castro, 2014; Liu et al., 2018). Bitcoin is commonly prevalent in developed and developing countries. Social media marketize the cryptocurrency and convey information about it to every social media user. Social trends also affect human psychology, whether positively or negatively; it is up to trending information (Tausczik and Pennebaker, 2010; Zhang et al., 2014; De Choudhury et al., 2016). Cultural factors can influence employees’ attitudes in implementing environment risk policies (Sarfraz et al., 2018).

The aspiring Bitcoin investors checked the cryptocurrency’s returns and decided to save them. A Bitcoin user might receive good incentives by using this virtual currency for various payments and investments. The investors, users, and researchers are required to know about the financial models and tools that measure Bitcoin. A comprehensive understanding of cryptocurrencies’ statistical properties will help them set the peculiarities and limitations of Bitcoin. This research analyzed dependent and independent variables and the long or short-run relationship between Bitcoin prices and COVID-19. The COVID-19 is used as a spreading pandemic, helping investors perceive Bitcoin prices’ behavior under any toxic disease breakout. The price trajectories of Bitcoin be influenced by economic and political events, government regulations, speculation of exchange, mutual influence, global news, and hacking threat, and these all areas explored by the researchers. This research will be interlinked with the relationship between Bitcoin price volatility and COVID-19. The nature of Bitcoin and COVID-19’s relationship will be explored, whether long-run or short-run. This research basically address three research questions which are mentioned below:

Is the COVID-19 affected the Bitcoin prices?
What is investor psychology’s impact on Bitcoin volatility during the initial era of COVID-19?
Is the COVID-19 affect the Bitcoin prices for a short or long period?

## Materials and Methods

The primary model of this research is given below:

$$\Delta \,B\,T\,P_{t}=\beta_{0}+\beta_{1}\,C\,D+\beta_{2}\,C\,C+\varepsilon_{t}$$

In the above equation, the sign of delta Δ used for the first difference while β_*0*_, β_*1*_, β_*2*_ are demonstrated the independent parameters. The BTP is Bitcoin prices, the CD is daily-confirmed deaths, and CC is daily-confirmed cases of COVID-19. The ε_*t*_ is an error term with respect to time.

### Data Description

This research analyzed Bitcoin prices’ daily data, cumulative confirmed cases, and deaths caused by COVID-19. The data set covers the initial period from January 20, 2020, to April 30, 2020. The Bitcoin prices are taken against the United States dollar. The data sets were extracted from different cites, i.e., the COVID-19 data was from the World Health Organization (WHO), and the Bitcoin data was from the coin desk official. This research was based on the initial period of COVID-19 breakout because it was an unexpected situation fly Bitcoin. After 30 April 2020, the executives have understood the situation and established their working technologies in their homes and restrictive offices working with the following SOPs.

### Methods

#### Unit-Root Test

The unit-root test verifies the variables’ integration order. The existing literature also supported the augmented Dickey–Fuller (ADF) test as the well-known test to check the variables’ integration order.

This research has also employed the ADF test for unit root through the following equation:

$$\Delta \,Y_{t}=\alpha +\beta_{t}+\rho \,Y_{t-1}+\sum_{i=1}^{k}\gamma_{i}\,\Delta \,Y_{t-1}+e_{t}$$

$$H_{0}=T\,h\,e\,e\,x\,i\,s\,t\,e\,n\,c\,e\,o\,f\,U\,n\,i\,t-R\,o\,o\,t\,i\,n\,v\,a\,r\,i\,a\,b\,l\,e\,s$$

$$H_{1}=T\,h\,e\,v\,a\,r\,i\,a\,b\,l\,e\,s\,a\,r\,e\,s\,t\,a\,t\,i\,o\,n\,a\,r\,y$$

Where ΔY_t_ = Y_t_−Y_t−1_;Y_t−1_ = Y_t−1_−Y_t−2_; Δ is the difference operator; α is the constant; β is the coefficient on-time trend *t*; ρ represents the number of lag, empirically determined using the Schwarz information criterion (SIC); and *e*_*t*_ is the error term with zero mean and variance. The coefficient term Y_t−1_ is later included in testing the coefficient’s significance (MacKinnon et al., 1999; Ntshangase et al., 2016; Naseem et al., 2019). The augmenting process is completed with the possible removal of autocorrelation among error terms. The null hypothesis of ADF states that the unit root exists in the data series, and the alternative hypothesis rejects the null by declaring the data series’ stationarity.

#### Co-integration Test

The co-integration test is employed after checking the data series’ stationarity or order of integration between variables. The co-integration test measures the long-run relationship or equilibrium through many time series datasets with a linear combination of variables. This research employed the Johansen co-integration test to check the stability and long-term equilibrium relationship between the variables by the following equation:

$$\Delta Y_{t}=\Pi_{t-1}+\sum_{i-1}^{p-1}\Gamma_{i}\Delta Y_{t-1}+Bx_{t}+{\text{μ}}_{t}$$

$$\Pi =\sum_{i-1}^{p}A_{i}-I,\Gamma_{i}=-\sum_{i=t+1}^{p}A_{j}$$

The above equation expresses Π as an indicator of the adjusted disequilibrium matrix. The stacking coefficient *A* boosted the endogenous factor’s speed of change in counter to disequilibrium. The sign Γ is used to capture the short-term dynamic adjustment (Otero and Smith, 2000; Yavuz, 2014; Naseem et al., 2019). This test progression can declare the association of variables with their positions in the matrix and featuring roots. The co-integration test’s null hypothesis is that the cointegration exists in the data series, and the alternative hypothesis is opposed.

#### Vector Error Correction Model

The vector error correction model (VECM) is used to determine the causality direction of variables after the confirmation of a cointegration relation (Sulaiman and Abdul-Rahim, 2018; Ren et al., 2020). The VECM framework was structured as follows:

$$\left[\begin{array}{c} \Delta \,l\,n\,C\,D-1_{t}\\ \Delta \,l\,n\,C\,C-2_{t} \end{array}\right]=\left[\begin{array}{c} \theta_{1}\\ \theta_{2} \end{array}\right]+\left[\begin{array}{c} d_{11\,m}\,d_{12\,m}\\ d_{21\,m}\,d_{22\,m} \end{array}\right]\times \left[\begin{array}{c} \Delta \,l\,n\,C\,D-1_{t-1}\\ \Delta \,l\,n\,C\,C-2_{t-1} \end{array}\right]\;+\cdots +\left[\begin{array}{c} d_{11\,n}\,d_{12\,n}\\ d_{21\,n}\,d_{22\,n} \end{array}\right]\times \left[\begin{array}{c} \Delta \,l\,n\,C\,D-1_{t-1}\\ \Delta \,l\,n\,C\,C-2_{t-1} \end{array}\right]\;+\left[\begin{array}{c} \lambda_{1}\\ \lambda_{2} \end{array}\right]\,\left(E\,C\,M_{t-1}\right)+\left[\begin{array}{c} \varepsilon_{1\,t}\\ \varepsilon_{2\,t} \end{array}\right]$$

Where the coefficients λ_1_−λ_2_ indicate the error correction term and the homoscedastic disturbance term denoted by ε_1*t*_−ε_2*t*_, and *ECM*_*t–1*_ represents the long-run equilibrium and speed of adjustment. In the equation, CD represents cumulative deaths, and CC represents cumulative confirmed cases of COVID-19.

#### Granger Causality Test

The following equation estimates the Granger causality test:

$$X_{t}=\alpha_{0}+\sum_{j=1}^{k}\alpha_{1\,s}\,X_{t-s}+\sum_{i=1}^{m}\alpha_{2\,i}\,Y_{t-m}+\varepsilon_{1\,t}$$

$$Y_{t}=\beta_{0}+\sum_{j=1}^{n}\beta_{1\,j}\,Y_{t-j}+\sum_{h=1}^{p}\beta_{2\,h}\,X_{t-h}+\varepsilon_{2\,t}$$

In the above equation, it is assumed that the terms ε_*1t*_ and ε_*2t*_ are not correlated, as *E*(ε_1*t*_,ε_2*t*_) = 0 = *E*(ε_2*t*_ε_2*s*_)…*s*≠*t*. The unidirectional causality from Bitcoin prices to death and confirmed cases of COVID-19 is expressed in this equation. The significance of α_*2i*_ and β_*2h*_ is confirmed by the mutual dependency of two specific variables and vice versa. The term Y and X will be independent if the α_*2i*_ and β_2*h*_ are not zero.

## Results

In Table 1, the ADF test results for integrating Bitcoin prices and COVID-19 are presented. The null hypothesis of a unit root in the series could not be rejected at a 10% level of significance, except for cumulative deaths. The dependent and independent variables are significant at a 1 and 5% level of significance. The Bitcoin prices and cumulative deaths are significant at a 1% level of significance, while cumulative confirmed cases are significant at a 5% level. After taking the first differences, both conditional series have shown a 1 and 5% level of significance for all variables. The last column has contained the order of integration in which I represent the integrated order of variables (Johansen, 1992; Cheung and Lai, 1995; Mohsin et al., 2020a; Salamat et al., 2020). The numeric values 0 and 1 are used for level and first difference. The table below shows I (1) in front of all causes because the results support the significance of the first difference, which is a fundamental requirement to run the cointegration test.

**TABLE 1:** Augmented Dickey-Fuller unit-root test.

Stationarity test				Order of co-integration
	
Variables	Unit Root Test	Augmented Dickey-Fuller Test (Constant)		ADF
			
		ADF *t*-Stat.	*P*-value	
BTC	Level data	–0.8913	0.7855	I (1)
	1st difference data	–9.4972	0.0000	
CD	Level data	–2.8088	0.0630	I (1)
	1st difference data	–12.8842	0.0000	
CC	level data	3.8711	1.0000	I (1)
	1st difference data	–3.4710	0.0123	

Table 2 contains the second step results in which the appropriate lag length is selected. Based on the Vector Autoregression lag order selection criteria, lag three is chosen for Bitcoin prices by three information criteria, i.e., the Akaike information criterion (AIC), SIC, and Hannan–Quinn information criterion (HQC). In contrast, in fatal accidents, AIC and HQC have chosen lag three (Mohsin et al., 2020b).

**TABLE 2:** VAR Lag order selection criteria.

Lag	Log L	LR	FPE	AIC	SC	HQ
0	−1723.89	NA	2.30E+18	50.79091	50.88883	50.82971
1	−1448.13	519.0892	8.98E+14	42.94485	43.33653	43.10005
2	−1435.03	23.49738	7.98E+14	42.82435	43.50979	43.09594
3	−1381.96	90.53527*	2.19e+14*	41.52811*	42.50730*	41.91609*

*Shows 1% level of significance.

After selecting the appropriate lag length, the next step is to check the long-run relationships among the variables used in this research. The Johansen cointegration test was employed to check the long-run relationship among variables, and the results are presented in Table 3. The results are in two sections, i.e., the Johansen max test and trace eigenvalue. The results indicated that the variables are co-integrated at 1 and 5% level of significance with values 140.4974 (None) and 14.87688 (At most 1), respectively, for maximum eigenvalue. The maximum eigenvalue value (trace) for Bitcoin is 155.5734, which shows the significance at a 1% level of significance. This research used the first equation of maximum eigenvalue and trace value because of commonalities. The first equation of both tests is significant at a 1% level of significance. The cointegration results confirmed the long-run relationship between Bitcoin prices and COVID-19. In the next step, the trace and Max-Eigenvalues test results generated a cointegration equation based on the log-likelihood ratio.

**TABLE 3:** Unrestricted co-integration rank test (Maximum Eigenvalue).

Hypothesized No. of CE(s)	Max-Eigen Statistic	Critical value (0.05)	Prob.
None*	140.4974	21.13162	0.0001
At most 1	14.87688	14.2646	0.04
At most 2	0.199096	3.841466	0.6554
**Unrestricted Co-integration Rank Test (Trace)**			
Hypothesized No. of CE(s)	Trace Statistic	Critical value (0.05)	Prob.
None*	155.5734	29.79707	0.0001
At most 1	15.07598	15.49471	0.0577
At most 2	0.199096	3.841466	0.6554

*Shows 1% level of significance.

A linear combination of Bitcoin prices and COVID-19 could be scrutinized from the cointegration equation. This cointegration equation is also used to verify long-run relationships, and it is confirmed for this research. The results of the cointegration equation are presented in Table 4. The normalized equation of Bitcoin prices expresses a significant positive relationship.

**TABLE 4:** Co-integration test.

1 Co-integrating equation(s)		Log-likelihood -511.2092
BTC	Cumulative Deaths	Cumulative Cases
1.000	180.8510	−2.158967
Standard Error	(−12.0147)	(−0.16688)

In Table 5, the VECM’s results are presented with multiple time series’ long-run and short-run relationships between dependent Bitcoin prices and COVID-19. A bidirectional relationship was observed between cumulative death cases and cumulative confirmed cases (*CD*_−1_⇔*CC*_−1_), with a difference at a 1% level of significance and unidirectional causality with a difference (*CD*_−2_ = > *CC*_−2_≠*CD*_−2_) also significant at a 1% level. The speed of adjustment from short-term equilibrium to long-term equilibrium for Bitcoin price is 2.09%, which is negatively insignificant. COVID-19 does not influence the Bitcoin prices for the long-term because conventional money is replaced by cryptocurrency. According to scientists and paramedical staff, the virus’s source is human beings. When it was declared that the exchange of conventional money is also a reason for the spread of the virus, governments suggested cashless transactions to avoid spreading it. The businessmen, students, and investors needed to pay for different trade, fees, and exchange purposes. So, Bitcoin came to use for every payment kind. The business moved to homes, and everything was stuck during the lockdown. Online payment is the best solution to avoid difficulties during a pandemic. At the beginning of the pandemic breakout, Bitcoin prices have shown a downward trend, but as people started the transactions online or Bitcoin, the upward trend is observed in Bitcoin prices.

**TABLE 5:** Vector error correction estimates test.

Error correction	Price	CD	CC	ECT_*t*–1_
D(PRICE(−1))		–0.0068	–0.1814	–0.0209
D(PRICE(−2))		–0.0180	–1.1179	
D(CD (−1))	6.6845		−171.8432*	0.0148*
D(CD(−2))	2.6652		−10.0428*	
D(CC(−1))	–0.0708	0.0491*		0.5549*
D(CC(−2))	–0.0272	0.0315		
C	–86.2452	−8.8489*	−612.879*	

*Shows 1% level of significance.

Table 6 is a summary of Table 5 and a crosscheck of directional causality. The VECM declared the long-run relationship among variables and the speed of variables’ adjustment from the short-run to the long-run equilibrium level. The total observations of the research are 69. The Granger causality test summary shows the Bitcoin price and cumulative death cases are unidirectional at a 5% level of significance. In comparison, the Bitcoin price and cumulative confirmed cases are bound in a unidirectional relation at the rate of 5% level of significance. The unidirectional positive significant relationship of Bitcoin price with cumulative death cases and confirmed cases declared that Bitcoin was affected by the pandemic but positively. The cumulative confirmed cases and deaths are interdependently related to each other. An increase in cumulative confirmed cases is why there is an increase in the cumulative death cases and vice versa. The ECT_*t*–1_ is negative but insignificant. The negative sign of ECT_*t*–1_ confirms the long-run relationship of variables with a positive effect.

**TABLE 6:** Granger causality test.

Direction of causality	Observations	F-Statistics	Prob.
Cumulative deaths does not granger cause price	69	0.6469	0.6922
Price does not granger cause cumulative deaths		2.9497**	0.0149
Cumulative cases does not granger cause price	69	0.7078	0.6447
Price does not granger cause cumulative cases		2.3919**	0.0408
Cumulative cases does not granger cause cumulative deaths	69	87.7901*	2.00E-25
Cumulative deaths does not granger cause cumulative cases		73.4563*	1.00E-23

*, ** shows 1, 5, and 10% level of significance, respectively.

## Discussion

Under the state theory of money, fiat money can spend on another good that the money holder must require. Money is a mode of exchange so that the state theory claims the worth of legal money. The cryptocurrency, i.e., Bitcoin is a form of fiat money in a finite economy. Additionally, Bitcoin is decentralized and without backed reserve money. It solely exists without the control of any single government or big financial company. At the starting period of Bitcoin, the investors and general public believed that it would vanish suddenly from the market same as its entrance. Nowadays, Bitcoin drives its worth due to the rapid increase in investment and other buying agents’ trust. The increasing value and trading transactions of Bitcoin declared that Bitcoin retains its value and never ends up with a so-called hot potato. The authenticity and trust-ability of Bitcoin were observed in the COVID-19 period. The virus used human beings as a transmitter resource, so the government banned conventional money, banking transactions, and automatic teller machine (ATM) transactions to avoid spreading viruses. The pandemic restricted people in the home but not activities of life. All payments, receipts, and trading activities needed a safe, decentralized, and quick payment mode for pending transactions. Bitcoin served as online, quick, safer, and decentralized money that eased the businesspersons, traders, and even the public. In the COVID-19 period, the graph of Bitcoin is shown an upward trend in both senses, i.e., prices and trading transactions. This research also supported the increasing trend of Bitcoin and the positive relationship between COVID-19 and Bitcoin returns. As a virtual currency, Bitcoin plays a crucial role in having the feature of online, virtual, cashless, and un-centralized currency. The stability of Bitcoin increases investors’ confidence and the users’ trust.

## Conclusion

This research’s primary focus was to examine Bitcoin prices’ volatility due to COVID-19 and people’s psychological behavior under strenuous circumstances. COVID-19 has further been categorized into two parts, i.e., cumulative deaths and cumulative confirmed cases. In this research, daily data were used from January 20, 2020, to April 30, 2020. The daily data set was used for accuracy, the impact of investors’ prices, and the changing of Bitcoin prices and investment decisions. The unwanted data has covered the initial period of COVID-19 due to checking the sudden effects of the pandemic on Bitcoin prices. The econometric methodology was used to measure the relationship between Bitcoin prices and COVID-19. The ADF test checked the data stationarity and confirmed that all data series were unit-root free at a 1% level of significance. The Johansen cointegration technique confirmed a positive and significant long-run relationship between Bitcoin prices and COVID-19. The VECM confirmed the directional causalities for the short-term.

The short-term negative significance relationship shows the negative impact of COVID-19 on Bitcoin prices. At the inception of COVID-19, the graph of Bitcoin prices showed a downward trend in every field of life. The positive long-run relationship between Bitcoin prices and COVID-19 has an interesting impact on the growth. Conventional money was banned due to the spread of the virus through conventional money, so people had trouble with different kinds of payments. As a virtual currency, Bitcoin plays a crucial role in having the feature of online, virtual, cashless, and un-centralized currency. The stability of Bitcoin increases investors’ confidence and the users’ trust. People’s psychological state has changed by using Bitcoin for payments and safely done their exchanges. This research declares pandemic (COVID-19) relationship and Bitcoin prices for decision-makers, the public, and practitioners, and the contribution of virtual currencies in psychological behavior. This research shows the path for a specific pandemic breakout, i.e., COVID-19 and the communicably spread epidemics. This research suggests that investors should fully aware of the international monetary market and policies because they are trading uncontrolled and decentralized assets. The digital, fiat or online money depends on the internet, and investors’ unavailability becomes the cause of loss. Bitcoin has a bundle of benefits, but at the same time unpredictability, it becomes the reason for bankruptcy. The possible policies and measures that might be influenced the prices and returns of Bitcoin can be explored in future research. This research is limited to the initial period of COVID-19. It can be comprehended with current data, and future research may be explored pre, during, and post era of COVID-19.

## Data Availability Statement

Publicly available datasets were analyzed in this study. This data can be found here: https://covid19.who.int/ and https://www.coindesk.com/.

## Author Contributions

All authors listed have made a substantial, direct and intellectual contribution to the work, and approved it for publication.

## Conflict of Interest

The authors declare that the research was conducted in the absence of any commercial or financial relationships that could be construed as a potential conflict of interest.
